# Targeting KIT on innate immune cells enhances the antitumor activity of checkpoint inhibitors in vivo

**DOI:** 10.1186/2051-1426-3-S2-O12

**Published:** 2015-11-04

**Authors:** Richard Gedrich, Scott Seibel, Theresa LaVallee, Joseph Paul Eder

**Affiliations:** 1 Kolltan Pharmaceuticals, Inc., New Haven, CT, USA; 2 Yale Comprehensive Cancer Center, New Haven, CT, USA

## 

Mast cell infiltrates are associated with tumors, though their role in the tumor microenvironment remains unclear. Mast cells express high levels of KIT throughout differentiation and as mature cells. Mast cells in tumors have been shown to release proinflammatory cytokines and promote angiogenesis, increasing tumor growth and metastasis. KIT signaling and mast cells also appear to modulate myleloid-derived suppressor cell (MDSC) development, recruitment and activity in tumors. Furthermore, treatment of tumor-bearing mice or cancer patients with small molecule KIT inhibitors or an anti-mouse KIT antibody decreased tumoral MDSCs and other immunosuppressive cells including regulatory T cells. KTN0158 is a humanized anti-KIT IgG1 monoclonal antibody that specifically binds KIT and is being developed as a potential therapy for cancer and mast cell-related diseases such as neurofibromatosis type 1 (NF1). It binds canine, feline, non-human primate and human KIT with high affinity, but does not bind mouse or rat KIT. KTN0158 inhibits KIT signaling and function in vitro and in vivo and has shown antitumor activity in dogs with mast cell tumors expressing either wild-type or mutant KIT. Immune checkpoint inhibitors targeting the CTLA-4 and PD-1 pathways have demonstrated single agent efficacy in cancer patients, and the combinations have shown superior efficacy in some preclinical and clinical settings. To test the effects of KIT inhibition on immune tolerance in syngeneic mouse tumor models, the anti-mouse KIT monoclonal antibody ACK2 was used as a surrogate for KTN0158 and tested alone and in combination with CTLA-4 and PD-1 inhibitors. In the Colon26 model, single agent treatment with the anti-KIT antibody had little antitumor activity. However, the combination of anti-KIT and anti-CTLA-4 had substantial antitumor effects, comparable to those observed for anti-CTLA-4 plus anti-PD1. These antitumor effects were dose-dependent. Pharmacodynamic assessments will also be presented. Collectively, these data suggest that the combination of immunotherapies targeting KIT on innate immune cells and checkpoint pathway inhibitors may have clinical benefit.

